# Cannabidiol Is a Potential Therapeutic for the Affective-Motivational Dimension of Incision Pain in Rats

**DOI:** 10.3389/fphar.2017.00391

**Published:** 2017-06-21

**Authors:** Karina Genaro, Débora Fabris, Ana L. F. Arantes, Antônio W. Zuardi, José A. S. Crippa, Wiliam A. Prado

**Affiliations:** ^1^Department of Neuroscience and Behavioral Sciences, Ribeirão Preto Medical School, University of São PauloSão Paulo, Brazil; ^2^Department of Psychology and Education, Faculty of Phylosophy, Science and Language Studies of Ribeirão Preto, University of São PauloSão Paulo, Brazil; ^3^National Institute of Science and Technology for Translational Medicine, Conselho Nacional de Desenvolvimento Cientifico e TecnologicoBrasília, Brazil; ^4^Department of Pharmacology, Ribeirão Preto Medical School, University of São PauloSão Paulo, Brazil

**Keywords:** endocannabinoids, cannabidiol, pain, allodynia, aversion, anterior cingulate cortex

## Abstract

**Background:** Pain involves different brain regions and is critically determined by emotional processing. Among other areas, the rostral anterior cingulate cortex (rACC) is implicated in the processing of affective pain. Drugs that interfere with the endocannabinoid system are alternatives for the management of clinical pain. Cannabidiol (CBD), a phytocannabinoid found in *Cannabis sativa*, has been utilized in preclinical and clinical studies for the treatment of pain. Herein, we evaluate the effects of CBD, injected either systemically or locally into the rACC, on mechanical allodynia in a postoperative pain model and on the negative reinforcement produced by relief of spontaneous incision pain. Additionally, we explored whether CBD underlies the reward of pain relief after systemic or rACC injection.

**Methods and Results:** Male Wistar rats were submitted to a model of incision pain. All rats had mechanical allodynia, which was less intense after intraperitoneal CBD (3 and 10 mg/kg). Conditioned place preference (CPP) paradigm was used to assess negative reinforcement. Intraperitoneal CBD (1 and 3 mg/kg) inverted the CPP produced by peripheral nerve block even at doses that do not change mechanical allodynia. CBD (10 to 40 nmol/0.25 μL) injected into the rACC reduced mechanical allodynia in a dose-dependent manner. CBD (5 nmol/0.25 μL) did not change mechanical allodynia, but reduced peripheral nerve block-induced CPP, and the higher doses inverted the CPP. Additionally, CBD injected systemically or into the rACC at doses that did not change the incision pain evoked by mechanical stimulation significantly produced CPP by itself. Therefore, a non-rewarding dose of CBD in sham-incised rats becomes rewarding in incised rats, presumably because of pain relief or reduction of pain aversiveness.

**Conclusion:** The study provides evidence that CBD influences different dimensions of the response of rats to a surgical incision, and the results establish the rACC as a brain area from which CBD evokes antinociceptive effects in a manner similar to the systemic administration of CBD. In addition, the study gives further support to the notion that the sensorial and affective dimensions of pain may be differentially modulated by CBD.

## Introduction

Pain is an experience that has somatosensory, affective, motivational and cognitive characteristics (Melzack and Casey, 1968), involves different brain regions and encompasses diverse neurochemical mechanisms (Bushnell et al., 2013). These mechanisms include multiple ascending spinal pathways to the brain, and this afferent circuitry is controlled by “top-down processing”. The anterior cingulate cortex (ACC) is a crucial component in an interconnected network of brain regions involved in pain perception, stress, anxiety, and reward (Etkin et al., 2011; Navratilova and Porreca, 2014; Zhang et al., 2017). ACC neurons connect with several regions important for pain processing, including the prelimbic, infralimbic and insular cortex, medial thalamus, amygdala, nucleus accumbens, and hippocampus (Strassels, 2006; Tracey and Mantyh, 2007; Vogt and Vogt, 2009; Neugebauer, 2015). The role of the rostral ACC (rACC) in the experience of pain has been confirmed in rodents (Johansen et al., 2001) and primates (reviewed in Shackman et al., 2011). Lesions of the ACC and cingulum bundle suppress emotional reactions of human patients to persistent pain (Foltz and White, 1962, 1968).

Considering the multidimensionality of pain, research within the last two decades has sought alternative treatment approaches, but the number of new drugs that have reached the stage of clinical trials has been still small (MacPherson, 2000). The endocannabinoid (eCB) signaling system regulates a broad spectrum of physiologic processes and has attracted considerable attention as a potential pharmaceutical target for modulating pain perception, emotional state, reward behaviors, learning and memory (Pacher et al., 2006; Bambico et al., 2007, 2009; Ahn et al., 2008; Di Marzo, 2008; Palazzo et al., 2010; Luongo et al., 2017).

Multiple biochemical pathways may participate in eCB formation (Di Marzo et al., 1994). Anandamide and 2-arachidonoylglycerol (2-AG) are the best-studied eCB isolated so far (Devane et al., 1992; Mechoulam et al., 1995) and produce their physiological effects by activating the cannabinoid CB1 and CB2 receptors (Matsuda et al., 1992; Munro et al., 1993; Kendall and Yudowski, 2017). These receptors have a characteristic distribution in the nervous system which is particularly enriched in cortex, hippocampus and amygdala, a distribution that corresponds to the most prominent behavioral effects of cannabinoids (Mackie, 2008). Although the eCB system plays an important role in nerve signal transduction at the central and peripheral levels, the lifespan of extracellular eCB is limited by a rapid and selective process of cellular uptake, which is accompanied by the actions of fatty acid amide hydrolase (FAAH) and monoacylglycerol lipase (MAGL), the primary hydrolytic enzymes for anandamide and 2-AG, respectively (Piomelli, 2005). For this reason, eCB appear to play a limited role, which might explain the difficulty of detecting effects of eCB in behavioral and neurochemical studies (Piomelli, 2003).

Phytocannabinoids found in *Cannabis sativa*, such as Δ^9^-tetrahydrocannabinol (THC) and cannabidiol (CBD) and synthetic cannabinoids – metabolically and chemically more stables – and inhibitors of eCB inactivation have been shown to enhance the action of eCB in reducing pain, inflammation, anxiety and depression in rodents with negligible changes in motility and behavior, as observed with direct CB1 agonists (Piomelli et al., 2006; Russo et al., 2007; Ahn et al., 2008; Petrosino and Di Marzo, 2010; Jensen et al., 2015; Kramer, 2015). Inhibitor of FAAH induces antidepressant-like effects in rodents (Gobbi et al., 2005) and genetic deletion of FAAH in mice confers resistance to anxiety-like and depression-like behavioral responses (Bambico et al., 2012). Results from preclinical and clinical studies suggest that CBD is an effective, safe, and well-tolerated drug (Russo and Guy, 2006). Although CBD is a negative allosteric modulator and displays low affinity for CB1 and CB2 receptors, it enhances eCB signaling in rodents through an inhibitory action on the mechanisms of eCB inactivation (i.e., the transporter and the FAAH enzyme) (Bisogno et al., 2001; Leweke et al., 2012). CBD has been considered a promising strategy against inflammatory diseases (Carrier et al., 2006) and neuropathic pain (Costa et al., 2007). However, the action of CBD on pain-induced affective-motivational changes has not been described. Notably, persistent pain conditions are often accompanied by emotional and cognitive disorders. These dysfunctional or maladaptive changes in aversive/motivational circuits likely contribute to the challenges of treating persistent pain.

The experimental approaches and methods used to measure the affective dimension of pain are still poor. A successful attempt has been made to assess pain as the opposite of pleasure, and its relief often promotes a positive emotional state that has been described as a reward (Fields, 1999; Leknes et al., 2008). Relief of pain aversiveness has been taken as a negative reinforcement that can be experimentally assessed by a conditioned place preference (CPP) paradigm (King et al., 2009; Navratilova et al., 2013, 2015). A deeper understanding of the neural basis of nociception and its aversive component could not only broaden our view on pain but also open new approaches to the management of acute and persistent pain states. The present study therefore evaluates the effect of CBD, injected either systemically or locally into the rACC, on mechanical allodynia in a postoperative pain model and on the negative reinforcement produced by relief of spontaneous incision pain. Additionally, we explored whether CBD underlies the reward of pain relief after systemic or rACC injection.

## Materials and Methods

### Animals

A total of 275 male Wistar rats from the animal housing facility of the University of São Paulo, Ribeirão Preto campus, weighing 220–240 g, were used. Rats were housed in groups of four per cage, with food and water available *ad libitum*, in a temperature-controlled room (23 ± 1°C) under an inverted 12 h/12 h light/dark cycle (lights on at 7:00 PM). The rats were transported to the experimental room in their home cages and left undisturbed for 1 h prior to testing. All efforts were made to minimize animal suffering and reduce the number of rats used. All of the experiments received formal approval from the Committee on Animal Research and Ethics, Ribeirão Preto Medical School, University of São Paulo (CEUA No. 0051/2016). The experiments reported in this article were performed in accordance with the recommendations of the Brazilian Society for Neuroscience and Behavior and complied with the United States National Institutes of Health Guide for the Care and Use of Laboratory Animals and the guidelines of the Committee for Research and Ethical Issues of the International Association for the Study of Pain (Zimmermann, 1983).

### Surgical Procedures

The rats were anaesthetized with ketamine/xylazine (100/7.5 mg/kg, i.p.) and fixed in a stereotaxic apparatus (David Kopf, Tujunga, CA, United States). The upper incisor bar was set 3.0 mm below the interaural line so that the skull was horizontal between the bregma and the lambda. After scalp anaesthetisation with 2% lidocaine, the skull was surgically exposed, and stainless-steel guide cannulae (12 mm length, 0.6 mm outer diameter, 0.4 mm inner diameter) were bilaterally implanted into the rACC using the bregma as the reference point (angle of 22°; coordinates: anterior/posterior, +1.8 mm; medial/lateral, ±1.8 mm; dorsal/ventral: –2.3 mm). Each cannula was fixed to the skull with dental cement and two stainless-steel screws. After surgery, each guide cannula was sealed with a stainless-steel wire to prevent obstruction. The rats then received an intramuscular injection of penicillin G benzathine (Pentabiotic, 600,000 IU, 0.2 ml; Fort Dodge, Campinas, SP, Brazil). After surgery, the rats were returned to their home cages in groups of four and were allowed to recover over a period of 5 days.

### Drug and Infusion Procedure

Cannabidiol (99,6% pure, kindly supplied by BSPG-Pharm, Sandwich, United Kingdom). The drug was dissolved in 2% TWEEN 80 (Sigma–Aldrich, St. Louis, MO, United States) and saline (NaCl 0.9%) for intraperitoneal injections and in 100% grape seed oil (Campos and Guimarães, 2008) for intracerebral injections. Lidocaine was obtained from Sigma–Aldrich. The rats received vehicle or CBD injections intraperitoneally or into the rACC. The doses and schedule of the injections were based on a previous study (Lemos et al., 2010). Infusions into the rACC were slowly delivered in a constant volume of 0.25 μL over 2 min using an infusion pump (Harvard Apparatus, Holliston, MA, United States) to minimize physical disruption of tissue at the injection site. A thin dental needle (0.3 mm outer diameter) attached via polyethylene tubing to a 5 μL Hamilton syringe was introduced through each guide cannula. The injection needle protruded 1.0 mm (rACC) below the ventral tip of the implanted guide cannula. The displacement of an air bubble inside a length of polyethylene tubing that connected the syringe to the injection needle was used to monitor the microinjections. A further 2 min were allowed for diffusion of the drug into the target structure before the injectors were removed.

### Incision Pain Model

Rats were anaesthetized with ketamine/xylazine (100/7.5 mg/kg, i.p.), and a 1 cm longitudinal incision was made through the skin of the right hind paw to expose the muscle, which was subsequently incised longitudinally as described elsewhere (Brennan et al., 1996). The incised skin was stitched with two 5-0 nylon sutures. The rats were tested 24 h after surgery.

### Algesimetric Test

The threshold for mechanical stimulation was assessed with an electronic von Frey apparatus (IITC Electronic Equipment, United States), which consisted of a rigid plastic tip (tip area = 0.7 mm^2^) connected to a hand-held probe unit. The rat was placed in an acrylic cage (12 cm × 10 cm × 17 cm) with a wire-grid floor for 30 min to allow behavioral acclimation to the environment. A tilted mirror below the grid provided a clear view of the animal’s hind paw. Increasing upward pressure was applied with the plastic tip against the mid-plantar surface of each hind paw, bordering the incision wound near the heel. During this procedure, the applied force in grams (g) was continuously recorded by a main unit connected to the probe. The threshold was determined by removal of the paw followed by clear flinching movements. At this moment, the movement of the probe stopped, and the intensity of the pressure at the threshold was automatically determined.

### Conditioned Place Preference

Experiments were conducted as described previously, using an unbiased conditioning protocol in which neither the apparatus (i.e., the CPP box) nor the procedure of animal assignment to the pairing chambers demonstrated preference before conditioning (Cunningham et al., 2003). On the preconditioning day (Day 1), rats were placed in the Place Preference System (Master-One Suprimentos and Equipamentos para Laboratório LTDA, Ribeirão Preto, SP, Brazil) consisting of a pair of chambers with distinct sensory cues and a neutral middle chamber from which the rats had free access to all chambers for 15 min (i.e., 900 s). The rats were monitored in the CPP boxes by video recorders, and the time spent in each chamber was evaluated by an investigator blind to the treatment. Rats that spent >720 s or <180 s in either testing chamber were excluded from the study (King et al., 2009). The rats were grouped to ensure that there was no average baseline chamber preference in any experimental group. On the morning of the conditioning day (Day 2), the rats were injected with vehicle or CBD either intraperitoneally or bilaterally into the rACC. One hour later, each rat received a saline injection into the right popliteal fossa and was immediately placed for 30 min in the chamber in which it spent more time in day 1; 4 h later, the rat received an injection of 4% lidocaine (0.3 mL) in the right popliteal fossa and was placed in the opposite conditioning chamber for 30 min. The saline and lidocaine administrations were performed under identical conditions. On the test day (Day 3), each rat was placed in the CPP box with free access to all chambers for 15 min, and the time spent in each chamber was recorded. Difference scores were calculated by subtracting the time spent in the drug-paired chamber on Day 1 (baseline) from that of Day 3 (testing).

### Histology

After the tests, the rats were deeply anaesthetized with a lethal dose of chloral hydrate (500 mg/kg intraperitoneally) and transcardially perfused with 0.9% saline followed by 10% formalin. The brains were removed and post-fixed in 10% formalin followed by a 10% formalin/30% sucrose solution until sectioning. Coronal brain sections (60 μm) were cut on a cryostat and wet mounted on glass microscope slides. Once dry, the sections were stained with cresyl violet (5%, Sigma–Aldrich) to visualize and identify microinjection sites by microscopic examination, according to the atlas of Paxinos and Watson (2006).

### Statistical Analysis

The software used for all statistical analyses was GraphPad Prism, version 6.0 (GraphPad Software, Inc., La Jolla, CA, United States). Withdrawal threshold (WT) data are expressed as the mean + standard error of the mean (SEM). Comparisons between vehicle and CBD groups were made by two-way repeated measures analysis of variance (ANOVA) to compare the groups across all timepoints. The factors analyzed were treatment, time and treatment × time interaction. *Post hoc* differences were tested using Dunnett’s multiple comparison tests. Comparisons between control and CBD groups on CPP tests were made by one-way ANOVA or unpaired *t*-test. *Post hoc* differences were tested using Tukey’s multiple comparison tests. Statistical analysis of the mechanical allodynia data of rats tested on CPP were analyzed by three-way repeated measures ANOVA to compare the groups across all timepoints. The factors analyzed were surgery (sham or incision), treatment (vehicle or CBD) and time. The level of significance was set at *p* < 0.05 in all cases.

## Results

### Systemic CBD Reduces Mechanical Allodynia in Injured Rats

**FIGURE 1:**
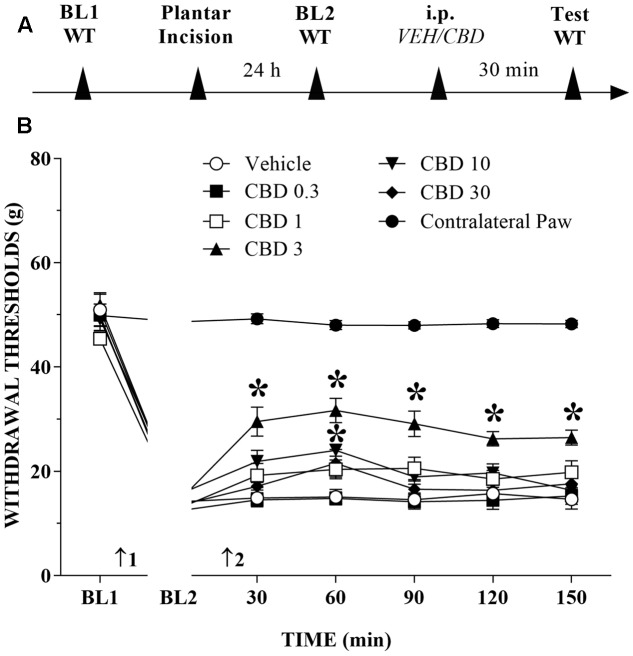
Reduction of tactile hypersensitivity by Cannabidiol (CBD). **(A)** Timeline of the protocol for the experiments; **(B)** The time-course of changes in the withdrawal thresholds (WT) measured in the operated and contralateral hind paws of the rats. Baseline (BL) was measured preoperatively (BL1) and 24 h postoperatively (BL2). Subsequently, the rat groups were treated with vehicle (VEH; *n* = 6) or CBD 0.3 to 30 mg/kg (*n* = 7–10), and WT was measured up to 150 min after injection. Arrows 1 and 2 indicate the times of surgery and intraperitoneal injection, respectively. Two-way ANOVA with Dunnett’s test: ^∗^*p* < 0.05 compared with vehicle. Data are means ± SEMs.

The timeline of the protocol for this experiment is shown in **Figure 1A**. Before incision of the hind paw, the withdrawal reflex was elicited by the application of approximately 50 g of force, using the electronic von Frey apparatus. In the incision pain model, we observed decreased mechanical thresholds, referred to as mechanical allodynia. The mean threshold measured 24 h after surgery decreased approximately 65% from the presurgical threshold, thus revealing the presence of mechanical allodynia. The systemic injection of CBD (0.3 to 30 mg/kg) produced a bell-shaped dose-related reduction of mechanical allodynia that lasted for at least 150 min at the 3 mg/kg dose. The decrease of mechanical allodynia was maximal at 60 min after the 3 and 10 mg/kg doses, which elicited a significant increase in the force required for paw withdrawal. The differences were statistically significant in terms of time [*F*_(6,534)_ = 524.0; *p* < 0.01], treatment [*F*_(6,89)_ = 149.2; *p* < 0.01] and the time × treatment interaction [*F*_(36,534)_ = 45.2; *p* < 0.01]. The threshold of the non-incised hind paw did not change throughout the period of observation (**Figure 1B**).

### Changes Induced by Systemic CBD Injections in the CPP Produced by Peripheral Nerve Block

**FIGURE 2:**
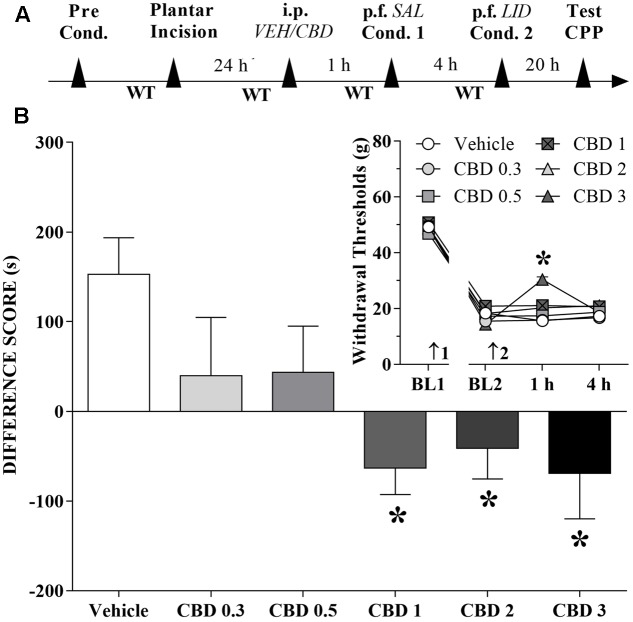
Cannabidiol may reduce (low doses) or invert (higher doses) the conditioned place preference (CPP) induced by peripheral nerve block. **(A)** Time line of the protocol for the experiments, **(B)** Peripheral nerve block produced significant CPP in incised rats pretreated intraperitoneally (i.p.) with vehicle (VEH; *n* = 16). CBD (0.3 and 0.5 mg/kg) reduced, and CBD (1 to 3 mg/kg) completely inverted, the CPP induced by peripheral nerve block (*n* = 9–12). One-way ANOVA with Dunnett’s test. The insert shows the time-course of changes in the WT measured in the operated hind paw of the rats. Arrows 1 and 2 indicate the times of surgery and injection, respectively. In rats with incisions, administration of vehicle and low doses of CBD had no effect on mechanical allodynia and its reversal by peripheral nerve block with lidocaine (LID) injected into the popliteal fossa (p.f.) of the injured limb. SAL, saline. Two-way ANOVA with Dunnett’s test: ^∗^*p* < 0.05 compared with vehicle. Data are means ± SEMs.

A time line of the protocol for the experiments is shown in **Figure 2A**. The motivational drive of rats with ongoing pain was assessed with the CPP paradigm. Rats that received CBD (1 to 3 mg/kg) before the first confinement did not present CPP by peripheral nerve block, but it seems that CBD itself produced CPP. The differences were statistically significant in terms of treatment [*F*_(5,63)_ = 3.8; *p* < 0.01] according to Dunnett’s test for CBD vs. vehicle in incised rats (**Figure 2B**). However, incision pain-produced mechanical allodynia in these groups was reduced only by intraperitoneal administration of CBD (3 mg/kg). The differences were statistically significant in terms of time [*F*_(3,189)_ = 857.6; *p* < 0.01], treatment [*F*_(5,63)_ = 4.7; *p* < 0.01] and interaction time × treatment [*F*_(15,189)_ = 5.4; *p* < 0.01] (insert of **Figure 2B**).

### Systemic CBD Induces CPP

**FIGURE 3:**
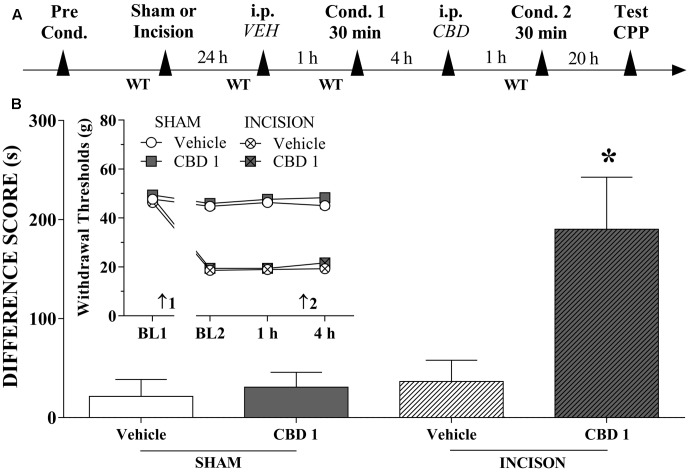
Cannabidiol induced significant CPP in incised but not sham rats. **(A)** Timeline of the protocol for the experiments; **(B)** CBD (1 mg/kg; *n* = 13) produced significant CPP only in incised rats. One-way ANOVA with Tukey’s test: ^∗^*p* < 0.05 compared with sham-incised groups and vehicle-treated incised group. Data are means ± SEMs. The insert shows that intraperitoneal (i.p.) administration of vehicle (VEH; *n* = 11) or CBD (1 mg/kg; *n* = 10) in sham-incised rats as well as vehicle (*n* = 12) in incised rats produced no significant effect on the WT. Arrows 1 and 2 indicate the times of surgery and injection, respectively.

We investigated whether systemically administered CBD could selectively activate reward circuits in injured rats. A timeline of the protocol for the experiments is shown in **Figure 3A**. CBD (1 mg/kg) induced significant CPP in injured rats [*F*_(3,42)_ = 6.2; *p* < 0.01]. CBD (0.5 mg/kg) induced no significant CPP in injured rats (not shown in Figures). CBD (1 mg/kg) did not induce CPP in sham rats (**Figure 3B**). One-way ANOVA with Tukey’s *post hoc* test demonstrated a significant effect of CBD (1 mg/kg) compared with all other groups (*p* < 0.05). Intraperitoneal administration of CBD (1 mg/kg) had no effect on paw WTs (insert in **Figure 3B**). There was a significant effect of time [*F*_(3,126)_ = 254.3; *p* < 0.01], treatment [*F*_(1,42)_ = 6.01; *p* < 0.01] and condition [*F*_(1,42)_ = 929.0; *p* < 0.01] but there was no interaction between three factors. These results suggest that CBD (1 mg/kg), which is not rewarding in sham-operated rats, become rewarding in injured rats, presumably because of pain relief.

### CBD Injection into the rACC Reduces Mechanical Allodynia in Incised Rats

**FIGURE 4:**
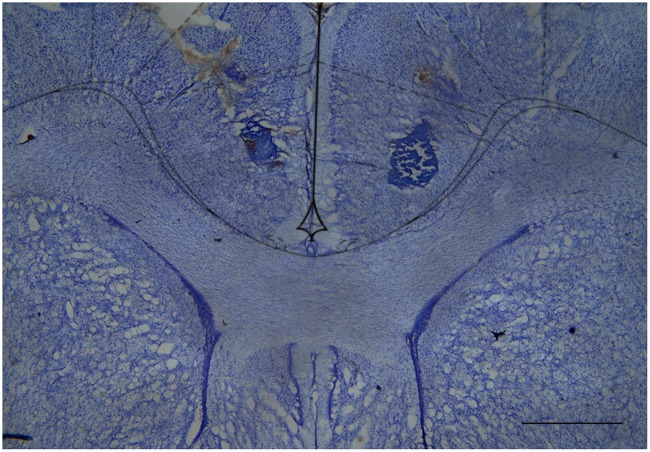
Cresyl violet-stained tissue showing the location of the cannula tip in the rostral anterior cingulate cortex. Scale bar = 1 mm.

**FIGURE 5:**
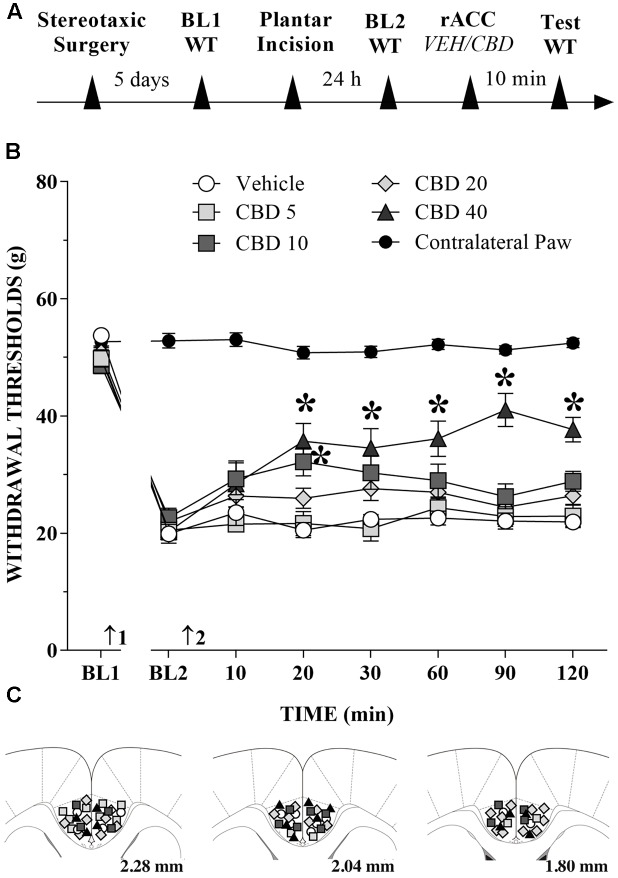
Reduction of tactile hypersensitivity by CBD. **(A)** Timeline of the protocol for the experiments; **(B)** The time-course of changes in the WT measured in the operated and contralateral hind paws of the rats. Baseline (BL) was measured preoperatively (BL1) and 24 h postoperatively (BL2). Subsequently, vehicle (VEH; *n* = 6) or CBD (5 to 40 nmol/0.25 μL; *n* = 7–11) was administered into the rostral anterior cingulate cortex (rACC), and WT was measured up to 120 min after injection. Arrows 1 and 2 indicate the times of surgery and injections into the rACC, respectively. Two-way ANOVA with Dunnett’s test: ^∗^*p* < 0.05 compared with vehicle. Data are means ± SEMs. Coronal sections taken from the atlas of Paxinos and Watson (2006) showing the locations of the injections of CBD in the rACC are shown in **(C)** using symbols as in the graph.

The great majority of injection sites were concentrated in the rACC (2.28 to 1.80 mm in relation to bregma). Representative photomicrograph of injection sites is shown in **Figure 4**. Time line of the protocol for the experiments is shown in **Figure 5A**. The local injection of CBD (40 nmol/0.25 μL) produced a reduction of the mechanical allodynia that lasted for at least 120 min. The decrease of mechanical allodynia started 20 min after CBD (10 and 40 nmol/0.25 μL) but was maximal at 90 min after the highest dose, which elicited a significant increase in the force required for paw withdrawal. The differences were statistically significant in terms of time [*F*_(7.476)_ = 133.4; *p* < 0.01], treatment [*F*_(5,68)_ = 134.1; *p* < 0.01] and interaction time × treatment [*F*_(35,476)_ = 17.2; *p* < 0.01]. The threshold of the non-incised hind paw did not change throughout the period of observation (**Figure 5B**). **Figure 5C** illustrates the microinjection sites in the rACC on diagrams of cross-sections from the atlas of Paxinos and Watson (2006).

### Changes Induced by CBD Injections into the rACC in the CPP Produced by Peripheral Nerve Block

**FIGURE 6:**
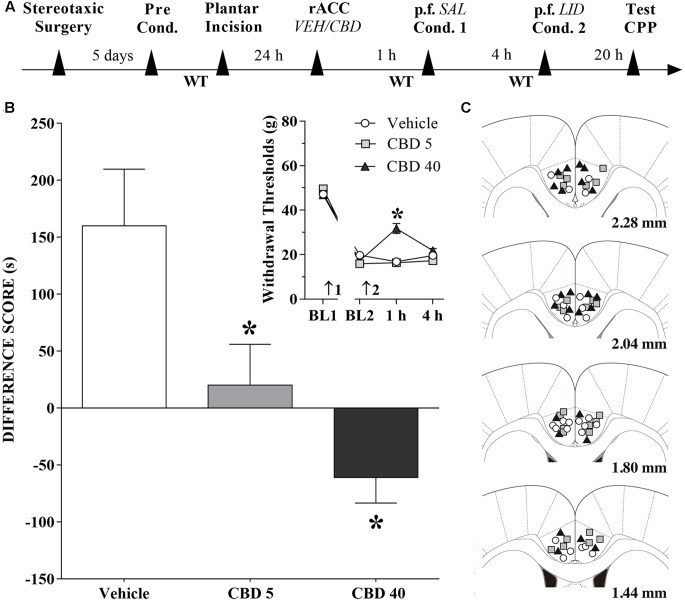
Cannabidiol into the rACC reduces (low doses) or inverts (higher doses) the CPP induced by peripheral nerve block. **(A)** Timeline of the protocol for the experiments; **(B)** Peripheral nerve block produced significant preference in incised rats pretreated intra-rACC with vehicle (VEH; *n* = 13). CBD (5 nmol/0.25 μL; *n* = 14) reduced, and CBD (40 nmol/0.25 μL; *n* = 12) inverted completely, the CPP by peripheral nerve block produced by injection of lidocaine (LID) into the popliteal fossa (p.f.). SAL, saline. One-way ANOVA with Dunnett’s test. The insert shows the time-course of changes in the WT measured in the operated hind paw of the rats. In rats with incisions, administration of vehicle and low doses of CBD had no effects on mechanical allodynia. CBD (40 nmol/0.25 μL) reduced mechanical allodynia. Arrows 1 and 2 indicate the times of surgery and injection, respectively. Two-way ANOVA with Dunnett’s comparison: ^∗^*p* < 0.05 compared with vehicle. Data are means ± SEM. Coronal sections taken from the atlas of Paxinos and Watson (2006) showing the location of the injections of CBD in the rACC are shown in **(C)** using symbols as in the insert.

A timeline of the protocol for the experiments is shown in **Figure 6A**. Intra-rACC injection of CBD (5 nmol/0.25 μL) reduced, and CBD (40 nmol/0.25 μL) inverted, CPP by peripheral nerve block (**Figure 6B**). The differences were statistically significant in terms of treatment [*F*_(2,36)_ = 8.2; *p* < 0.01], Dunnett’s test for CBD vs vehicle in incised rats. The effects of CBD (5 and 40 nmol/0.25 μL) injected into the rACC on mechanical threshold were also evaluated. Incision pain-produced mechanical allodynia was reduced only by administration of CBD at 40 nmol/0.25 μL (insert in **Figure 6B**). The differences were statistically significant in terms of time [*F*_(3,108)_ = 421.3; *p* < 0.01], treatment [*F*_(2,36)_ = 10.7; *p* < 0.01] and interaction time × treatment [*F*_(6,108)_ = 14.4; *p* < 0.01]. **Figure 6C** illustrates the microinjection sites in rACC on diagrams of cross-sections from the atlas of Paxinos and Watson (2006).

### CBD Injections into the rACC Induce CPP

**FIGURE 7:**
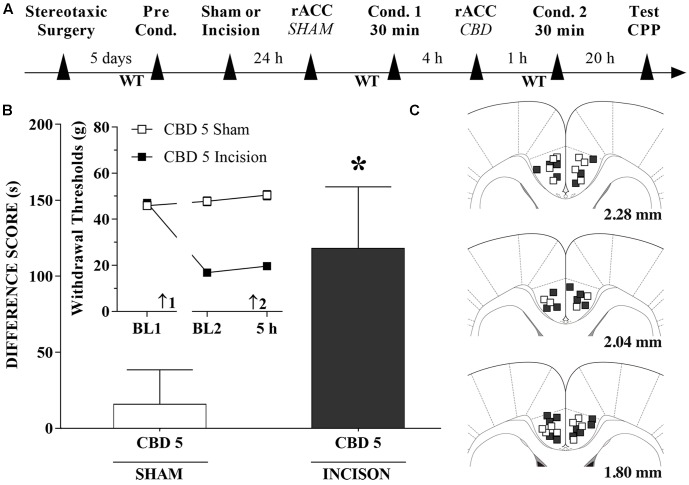
Cannabidiol injection into the rACC induced significant CPP in incised rats. **(A)** Timeline of the protocol for the experiments; **(B)** CBD at 5 nmol/0.25 μL produced significant CPP in incised rats (*n* = 12) but not in sham-incised rats (*n* = 10). One-way ANOVA with Dunnett’s comparison: ^∗^*p* < 0.05 compared with sham. Data are means ± SEM. The insert shows that CBD injected into the rACC in incised rats produced no significant effect on paw WTs. Arrows 1 and 2 indicate the times of surgery and injection, respectively. Coronal sections taken from the atlas of Paxinos and Watson (2006) showing the location of the injections of CBD in the rACC are shown in **(C)** using symbols as in the insert.

A timeline of the protocol for the experiments is shown in **Figure 7A**. CBD (5 nmol/0.25 μL), induced significant CPP in injured rats [*t*_(20)_ = 2.1; *p* < 0.05] (**Figure 7B**). The insert shows that CBD (5 nmol/0.25 μL) injected into the rACC had no effect on WTs 1 h after injections. There was a significant effect of time [*F*_(2,40)_ = 57.1; *p* < 0.01], treatment [*F*_(1,20)_ = 293.6; *p* < 0.01] and interaction time × treatment [*F*_(2,40)_ = 86.5 *p* < 0.01]. **Figure 7C** illustrates the microinjection sites in the rACC on diagrams of cross-sections from the atlas of Paxinos and Watson (2006).

## Discussion

The mechanical threshold of the incised paw was significantly decreased 24 h after a surgical incision but was not significantly changed in the contralateral side, as reported elsewhere (Brennan et al., 1996). The allodynic state was significantly less intense after an intraperitoneal injection of CBD (3 mg/kg), with the effect lasting for at least 150 min, while CBD (10 mg/kg) produced a significant anti-allodynic effect only 60 min after the injection. In contrast, CBD (0.3, 1, and 30 mg/kg) produced nonsignificant effect. Such bell-shaped dose-response curve have already been reported in the literature, as a characterizing the anxiolytic effect of systemic (Guimarães et al., 1990) and intracerebral (Campos and Guimarães, 2008) injection of CBD or systemic injection of other cannabinoids (Moreira et al., 2006).

The analgesic property of CBD is still controversial. Oral administration of CDB at doses of 200 mg/kg (Sanders et al., 1979) or 20 to 320 mg/kg (Sofia et al., 1975) is not active in mouse acetic acid-induced writhing test but oral CBD (0.1 mg/kg) is analgesic in mouse phenyl-*p*-benzoquinone-induced writhing test (Formukong et al., 1988; Evans, 1991). A single intraperitoneal injection of CBD (5 mg/kg) also did not change the nociceptive behavour of rats in the formalin test (Finn et al., 2004). The daily oral administration of CBD (2.5 to 20 mg/kg) from day 7 to 14 after the chronic constriction injury of the sciatic nerve, but not an acute oral dose of CBD (20 mg/kg), reduced the neuropathic pain of rats in a time- and dose-dependent manner (Costa et al., 2007). Systemic (10 to 50 mg/kg) or intrathecal (3 to 50 μg) administration of CBD suppresses chronic inflammatory and neuropathic pain in rodents (Xiong et al., 2012).

Hind paw incision in rats reduces the threshold of A∂- and C-fibers (Hamalainen et al., 2002), activates dorsal horn cells and induces central sensitization (Vandermeulen and Brennan, 2000). Moreover, postsurgical pain induces anxiety-like behavior that persists longer than hypersensitivity to mechanical stimulation (Li et al., 2010). The second experiment explored the CBD effects on pain relief, which is fundamentally rewarding and it is achieved by termination of an aversive event. Our data also confirmed that a hind paw incision provokes an aversive stimulus that persist for at least 2 days post-injury (Dai et al., 2011). This can provide a discriminative learning and fit easily within a reinforcement learning framework by aversive stimulus (King et al., 2009).

The presence of ongoing pain in our experimental conditions was confirmed by the demonstration of CPP following reward produced by peripheral nerve block, as proposed elsewhere (Navratilova et al., 2012). Intraperitoneal CBD (1 and 2 mg/kg) did not change, and CBD (3 mg/kg) significantly reduced the pain evoked by mechanical stimuli. However, CBD (1 to 3 mg/kg) inverted the CPP produced by peripheral nerve block. In contrast, intraperitoneal CBD at doses noneffective against incision allodynia (0.3 and 0.5 mg/kg) non-significantly reduced the CPP produced by peripheral nerve block. Therefore, the negative reinforcement produced by relief of ongoing pain with the peripheral nerve block is prevented in rats treated systemically with CBD even at doses that do not change the pain evoked by mechanical stimuli. It is worth further note that the local anesthetic may act as a negative reinforcement, however, the leg paralysis elicited by lidocaine may also act as a positive punishment. It supports the excitatory-inhibitory opponent relationship between rewards and punishments. In this case, beyond CBD does not promote motor paralysis it can acting to reduce the aversive state generated by the incision pain.

The absorption and distribution to brain of CBD after systemic administration in rats is relatively rapid but its apparent elimination half-life is 4 to 24 h (Deiana et al., 2012). The possibility remains that CBD may reduce spontaneous pain by itself once this drug was administered 5 h before the peripheral nerve block. The third experiment demonstrated for the first time that intraperitoneal CBD at doses that did not alleviate mechanical allodynia (0.5 and 1 mg/kg) elicited CPP in incised rats. In contrast, CBD (1 mg/kg) did not evoke CPP in sham-incised rats, thus agreeing with former reports that CBD (5 mg/kg) (Parker et al., 2004) and (10 mg/kg) (Vann et al., 2008) did not produce CPP in non-injured rodents. These results suggest that a dose of CBD that is not rewarding in sham-operated rats becomes rewarding in injured rats, presumably because of pain relief or reduction of pain aversiveness.

Although several brain structures contribute to pain and emotion processing, the circuits that engage the cingulate cortex are consistently activated in acute (Apkarian et al., 2005) and neuropathic (Hsieh et al., 1995) pain studies, and is involved in the affective dimension of pain (Rainville et al., 1997). The rACC have long been considered an important limbic component for encoding the emotional and motivational aspects of pain. In fact, chemical ablation of the rACC reduces pain affect in a rat formalin-induced CPP paradigm (Devinsky et al., 1995; Johansen et al., 2001; Johansen and Fields, 2004). Lesions of the ACC reduce pain unpleasantness in chronic pain patients (Foltz and White, 1962; Hurt and Ballantine, 1974). In agreement with an antihyperalgesic role for ACC ablation, electrical stimulation at most sites within the rat rACC facilitates the tail-flick reflex (Calejesan et al., 2000).

Considering the effects of systemic administration of CBD, we have also provided evidence that injection of CBD (40 nmol/0.25 μL) into the rACC produced a long-lasting (at least 120 min) reduction of mechanical allodynia in incised rats, whereas CBD (5 and 20 nmol/0.25 μL) were non-effectives. In addition, the smaller dose of CBD reduced and the higher dose inverted the CPP induced by peripheral nerve block. Therefore, negative reinforcement produced by relief of ongoing pain occurs in rats treated with CBD in the rACC even at a dose that does not prevent the mechanical allodynic state.

It should be noted that given its rich functional connectivity, the ACC is likely to be an important node in a complex network of brain structures that regulate this generalized enhancement in pain aversion (Porreca and Navratilova, 2017; Zhang et al., 2017). Nevertheless, ACC neurons project to or receive inputs from several regions important for pain processing, such as amygdala and nucleus accumbens (for review see Neugebauer, 2015). The central and basolateral nuclei of the amygdala, also participate in both pain- and fear-related negative emotion (for review see Gao et al., 2004), and contribute to the antinociceptive effect of systemically administered morphine (Manning, 1998). Additionally, CB1-immunoreactive cell bodies and fibers were demonstrated in cortical areas, amygdala and nucleus accumbens (Tsou et al., 1998; Moldrich and Wenger, 2000). Therefore, further studies are needed to investigate the effects of CBD on other brain structures involved in the aversiveness of spontaneous post-surgical pain.

The presented results have also shown for the first time that injection of CBD (5 nmol/0.25 μL) into the rACC, i.e., a dose that did not alleviate mechanical allodynia, elicited CPP in incised rats but not in sham-incised rats. Therefore, CBD injection into the rACC is itself able to relieve the aversiveness of ongoing incision pain.

ACC neurons responding to noxious stimulation were demonstrated in several animal species, including man, and they encode and transmit information related to the aversiveness of noxious stimuli (Sikes and Vogt, 1992; Yamamura et al., 1996; Koyama et al., 1998; Hutchison et al., 1999). Lesions of the rACC prevent avoidance learning elicited by tonic noxious stimuli (Johansen et al., 2001) and eliminate the aversiveness of spontaneous neuropathic pain (Qu et al., 2011). Neuroimaging studies have found evidence for both an anxiolytic effect of CBD and a critical modulatory role of the ACC in the effects of CBD (Fusar-Poli et al., 2009, 2010). The idea that emerged from our results is that CBD could decrease the activity of rACC, which would be in line with the anxiolytic effect of CBD in humans that is correlated with decreased activation in the ACC (Fusar-Poli et al., 2009). In addition, neurons and fibers with CB1-like immunoreactivity have already been identified in the cingulate cortex (Tsou et al., 1998; Moldrich and Wenger, 2000). CBD does not have significant intrinsic activity on CB1/CB2 receptors (Howlett et al., 2002) but displays a partial antagonistic effect at these receptors (Thomas et al., 2007; Pertwee, 2008) and acts as agonist at the 5-HT1A subtype of serotonin receptor (Campos and Guimarães, 2008; Gomes et al., 2011; Rock et al., 2012) and the TRPV1 subtype of transient receptor potential vanilloid 1 (Bisogno et al., 2001), and it also interferes with adenosine uptake (Carrier et al., 2006) and nuclear receptors of the PPAR family (O’Sullivan and Kendall, 2010; Esposito et al., 2011). Whatever the mechanism involved, these data lead us to suggest that CBD acts to reduce the pronociceptive role of rACC, thus reducing aversion to ongoing pain (low doses of CBD) and mechanical allodynia of the incised hind paw (high doses of CBD).

The presented results resemble those reported by Navratilova et al. (2015) showing that systemic administration of morphine at a dose that did not reverse tactile allodynia induced CPP in spinal nerve-ligated but not sham rats, while a higher dose that produced a full reversal of tactile allodynia elicited CPP in both sham and spinal nerve-ligated rats. The injection of morphine into the rACC also produced CPP but did not influence evoked mechanical allodynia in incised rats (Navratilova et al., 2015). The non-steroidal anti-inflammatory drugs ketorolac and naproxen, commonly used for treatment of post-surgical pain, increase the release of dopamine from the nucleus accumbens (Xie et al., 2014), a finding consistent with CPP (Navratilova et al., 2012). The doses used, however, also reduced the post-incision allodynia evoked by tactile stimuli and, therefore, it is not clear yet if those drugs are effective to reduce spontaneous post-surgical pain.

Our findings may not be enough to indicate CBD for the management of ongoing postoperative pain. However, the similarity of the effects of low doses of CBD and morphine may somewhat strengthen the case for a combination of these drugs as an alternative for the management of postoperative pain. In addition, the action of CBD involves brain substrates that contribute to pain suppression, presumably by reducing the distress that accompanies pain, a phenomenon referred to as “affective analgesia” (Franklin, 1998). Because emotions are involved in the conceptualization, assessment, and treatment of persistent pain, this study opens the possibility that CBD may have translational value to human pain conditions where affective dimensions appear to be most relevant (Price, 2000) and may be considered as a clinical alternative to eliminate or attenuate negative emotions that accompany persistent pain.

## Conclusion

The present study has shown for the first time that CBD injected either systemically or into the rACC induces a long-lasting anti-allodynic effect with a bell-shaped dose-response curve in a rat model of incision pain. CBD injected systemically or into the rACC at doses that did not change the incision pain evoked by mechanical stimulation significantly reduced peripheral nerve block-induced CPP and produced CPP by itself. The study provides evidence that CBD influences different dimensions of the response of rats to surgical incision pain. The results establish the rACC as a brain area from which CBD evokes antinociceptive effects in a manner similar to the systemic administration of CBD. The study gives further support to the notion that the sensorial and affective dimensions of pain may be differentially modulated by CBD.

## Ethics Statement

All of the experiments received formal approval from the Committee on Animal Research and Ethics, Ribeirão Preto Medical School, University of São Paulo (CEUA No. 0051/2016). The experiments reported in this article were performed in accordance with the recommendations of the Brazilian Society for Neuroscience and Behavior and complied with the United States National Institutes of Health Guide for the Care and Use of Laboratory Animals and the guidelines of the Committee for Research and Ethical Issues of the International Association for the Study of Pain.

## Author Contributions

KG and WP have contributed to conception, drafting and revising the manuscript; DF and AA have conducted behavioral experiments; AZ and JC have contributed revising the manuscript.

## Conflict of Interest Statement

AZ and JC are co-inventors (Mechoulam R, JC, Guimaraes FS, AZ, JH, Breuer A) of the patent “Fluorinated CBD compounds, compositions and uses thereof. Pub. No.: WO/2014/108899. International Application No.: PCT/IL2014/050023”; Def. US no. Reg. 62193296; 29/07/2015; INPI em 19/08/2015 (BR1120150164927). University of São Paulo licensed it to Phytecs Pharm (Resolução USP No. 15.1.130002.1.1). University of São Paulo has an agreement with Prati-Donaduzzi (Toledo, Brazil): *“Desenvolvimento de um produto farmacêutico contendo canabidiol sintético e comprovação de sua segurança e eficácia terapêutica na epilepsia, esquizofrenia, doença de Parkinson e transtornos de ansiedade”*. JC received a travel support from BSPG-Pharm. The other authors declare that the research was conducted in the absence of any commercial or financial relationships that could be construed as a potential conflict of interest.
